# “Puberty age gap”: new method of assessing pubertal timing and its association with mental health problems

**DOI:** 10.1038/s41380-023-02316-4

**Published:** 2023-12-05

**Authors:** Niousha Dehestani, Nandita Vijayakumar, Gareth Ball, Sina Mansour L, Sarah Whittle, Timothy J. Silk

**Affiliations:** 1 Deakin University, Centre for Social and Early Emotional Development, School of Psychology, Faculty of Health, Geelong, VIC Australia; 2 Department of Psychiatry, Melbourne Neuropsychiatry Centre, The University of Melbourne and Melbourne Health, Melbourne, VIC Australia; 3 Centre for Adolescent Health, Murdoch Children’s Research Institute, Parkville, VIC Australia; 4 Department of Paediatrics, The University of Melbourne, Melbourne, Australia; 5 Department of Biomedical Engineering, The University of Melbourne, Melbourne, VIC Australia

**Keywords:** Psychiatric disorders, Predictive markers

## Abstract

Puberty is linked to mental health problems during adolescence, and in particular, the timing of puberty is thought to be an important risk factor. This study developed a new measure of pubertal timing that was built upon multiple pubertal features and their nonlinear changes over time (i.e., with age), and investigated its association with mental health problems. Using the Adolescent Brain Cognitive Development (ABCD) cohort (*N* ~ 9900, aged 9–13 years), we employed three different models to assess pubertal timing. These models aimed to predict chronological age based on: (i) observed physical development, (ii) hormone levels (testosterone and dehydroepiandrosterone [DHEA]), and (iii) a combination of both physical development and hormones. To achieve this, we utilized a supervised machine learning approach, which allowed us to train the models using the available data and make age predictions based on the input pubertal features. The accuracy of these three models was evaluated, and their associations with mental health problems were examined. The new pubertal timing model performed better at capturing age variance compared to the more commonly used linear regression method. Further, the model based on physical features accounted for the most variance in mental health, such that earlier pubertal timing was associated with higher symptoms. This study demonstrates the utility of our new model of pubertal timing and suggests that, relative to hormonal measures, physical measures of pubertal maturation have a stronger association with mental health problems in early adolescence.

## Introduction

Adolescence is a pivotal stage of development that involves a complex interplay of biological, emotional, cognitive, and behavioral changes. These multifaceted transformations are crucial for facilitating the transition from childhood to adulthood [1, 2]. Individuals also progress through puberty, the process of attaining reproductive maturity, during this period. This includes hormonal and physical changes such as body hair growth and gonadal maturation (along with the development of breasts and onset of menstruation in females) [3, 4]. The progression through puberty has been associated with an increase in susceptibility to a range of internalizing and externalizing problems [4–6], which may reflect the effects of hormones on the central nervous system and/or psychosocial mechanisms related to physical differences from peers [7]. Therefore, investigation of individual differences in pubertal processes that consider both hormonal and physical changes may lead to a better understanding of adolescent mental health problems.

While all typically developing individuals progress through the same stages of puberty (based on observable physical changes), the onset and speed of progression can differ across individuals. As such, at any given period during adolescence, there is marked variability in the pubertal stage, termed “pubertal timing” [8, 9]. Importantly, it is pubertal timing—not pubertal stage—that has been shown to be linked to the emergence and severity of mental health problems [10], though there are inconsistencies in the literature. A number of studies have found that in females, earlier pubertal timing is associated with internalizing problems [11, 12], including depression [13, 14], anxiety [15], eating disorders [16], and externalizing behaviors. Similarly in males, both early and late timing have been related to internalizing [11, 16] and externalizing problems [17, 18], while others have failed to identify any associations [9, 15]. Null and inconsistent findings in the literature may be due to small sample sizes and methodological differences between studies, with variation in the measures used to calculate pubertal timing likely important. Indeed, a meta-analysis of 101 studies found that the method of measuring puberty had a moderating role in the relationship between pubertal timing and mental health problems [5].

Different statistical approaches exist to measure the relative pubertal timing of individuals compared to same-aged peers [19]. A common approach is to regress age from pubertal status (e.g., based on Pubertal Developmental Scale [PDS] scores), with residuals reflecting earlier or later maturity relative to the group average [20, 21]. However, such approaches only capture individual differences in observable physical development and do not inform us about underlying biological mechanisms that are more reflected in hormone levels [22]. Hormone levels provide valuable information regarding the endocrine processes of puberty [23, 24]. Previous studies have also demonstrated associations between changes in hormone levels and adolescent mental health problems. For example, increasing levels of dehydroepiandrosterone (DHEA) have been linked to internalizing symptoms [25], while testosterone has been associated with externalizing and disruptive behaviors [26]. To the best of our knowledge, only one study has calculated pubertal timing with hormone data to predict internalizing behaviors in a sample of 174 females [27]. Although the study did not find any significant associations, it is important to investigate this in a larger sample, of both females and males and to assess associations across different dimensions of mental health problems. Moreover, existing literature has highlighted diverse pathways through which the link between pubertal timing and mental health may operate. For instance, adolescents experiencing more physical development compared to their peers may encounter psychosocial challenges related to self-esteem and social interactions, potentially leading to feelings of loneliness that can lead to the development of internalizing behaviors [7] as well as a tendency to seek social connections with older age groups that increases exposure to opportunities to engage in risk-taking behaviors [7, 20]. Conversely, hormonal changes can exert influences on various brain regions [23–25] and neurotransmitter systems, such as the dopaminergic pathway [23], which can subsequently impact mental health [7, 20].

In this study, we propose a multivariate method to calculate pubertal timing that incorporates both observable physical changes as well as hormonal changes. This method draws upon the “brain age” approach [28, 29], where an association between multiple neuroimaging variables and chronological age is learned with supervised machine learning methods. Subtracting chronological age from brain age yields a “brain age gap” that reflects brain maturation relative to the group average [26, 28]. The major benefits of this model include being able to combine multiple features and reducing complex multivariate information to a single parameter. We propose to use a similar model to calculate estimates of pubertal timing by combining multiple puberty-related features. In contrast, existing strategies often focus on single features that only capture specific aspects or mechanisms of puberty, or they calculate the mean of multiple features that may obscure the relative importance of each one. The second benefit of this method is that it can model nonlinear relationships between multiple pubertal features and chronological age, which is important given that nonlinear associations between specific pubertal features of puberty and age have been observed in previous studies [30, 31].

Thus, the primary objective of this study was to develop a normative model of pubertal timing that integrates multiple measures encompassing hormone levels and physical changes. Normative modeling enables the study of pubertal information against age within the general population, while also allowing for the examination of individuals who deviate from established norms. To ensure robust model performance, we employed a rigorous cross-validation approach. We compared the performance of our “combined” normative model with that of a conventional linear model, which involves regressing age linearly from the total score of the pubertal features. Additionally, we compared performance with two unimodal models using a similar supervised machine learning approach: one utilizing hormonal measures alone and the other relying solely on physical measures. Our hypothesis postulated that the “combined” normative model (incorporating both hormonal and physical measures) would yield more accurate age predictions compared to the traditional linear model or unimodal normative models of puberty. Our second objective was to investigate the association between each pubertal timing model and multiple dimensions of mental health problems. We hypothesized that early pubertal timing would correlate with heightened mental health problems in both females and males, as suggested by a recent meta-analysis. Specifically, significant effects of comparable magnitude were expected for internalizing and externalizing problems, while attention problems were not anticipated to exhibit significant effects [5, 18]. Given the inconsistent findings on sex differences in prior research, we did not propose specific hypotheses.

## Materials and methods

To promote reproducible open research practices, all analyses conducted as part of this article are made publicly available in a Git repository (“https://github.com/Niousha-Dehestani/Puberty-age”). In addition, more detail about participants, measurements, and analysis is provided in the supplementary materials.

### Participants

Participants were drawn from the ongoing, longitudinal, Adolescent Brain Cognitive Development (ABCD) Study (https://abcdstudy.org/). Data was collected from ~11,500 children at baseline (47% females, aged 9–10 years old) from 21 sites across the United States, with annual data collection thereafter (see Supplementary Table 1 for demographic information). Data from the baseline to the 3rd annual follow-up waves (Release 4.0) were used in the current analyses. We excluded participants who had a mismatch between their biological sex (collected per visit with the salivary sample) and their self-reported gender, as well as those with missing data for biological sex (see Supplementary Information (SI) Appendix S1 for details of data cleaning procedures). The exact sample sizes utilized in each analysis are reported in detail below.

### Measures

#### Pubertal Development Scale

The Pubertal Development Scale (PDS) measures observable physical signs of puberty. It includes items on height, body hair, and skin change in both sexes, “already complete”). Onset of menarche was a binary variable (yes/no response) that was converted to one for “no” and four for “yes”. The PDS can either be collected via self- or parent-report. However, each of these measures has its own limitations. Parent report has good correspondence to clinician ratings, though this correspondence is lower in males [32]. Conversely, self-reporting is less accurate for individuals who are in the lower or upper pubertal stages as they tend to report toward the mid stages. Therefore, some studies have recommended using the parent-report PDS, especially in late childhood or early adolescence [33], and accordingly, the current study utilized this version. In this study, we used individual PDS items rather than the average PDS score typically used in prior literature [34]. This allowed us to flexibly model the contribution of each item in normative models, consistent with previous literature that has found that separate PDS items exhibit differential relationships with, e.g., brain structure [34].

#### Hormones

DHEA and testosterone (TST) levels were measured via salivary hormone samples assayed by Salimetrics. Although estradiol was also measured, it was not used in the current analyses due to it only being available for females and having excessive missingness (*n* = 780). The hormone data was cleaned based on the protocol that was published recently [35], which involved removing the confounding effects of collection time, duration of collection, wake-up time on collection day, having exercised before collection, and caffeine intake (with a linear mixed effect model, see SI for more details). Further information on the reliability and overall quality of the hormone data can be found in recent work [32].

#### Body Mass Index (BMI)

BMI was calculated as the average of two weight and height measurements per visit, assessed by the researcher. Next, BMI standard deviation scores (BMI *z*-scores) were calculated relative to age and sex, with reference to the CDC 2000 Growth Charts [36].

#### Sociodemographic variables

Five categories of race/ethnicity were coded: White, Black, Hispanic, Asian, and Other/Multi-race. Additionally, household income and education were obtained as measures of family socioeconomic status (SES) that were collected in a parent report.

#### Mental health problems

The Child Behavior Checklist (CBCL) (age 6–18 form [37]) was used to measure parent-reported mental health problems. CBCL includes eight syndrome scales; Anxious/Depressed, Withdrawn/Depressed, Somatic Complaints, Social Problems, Thought Problems, Attention Problems, Aggressive Behavior, and Rule-breaking Behavior as well as three broad summary scales of externalizing, internalizing, and total problems.

### Statistical analysis

#### Calculating pubertal timing

To calculate the pubertal timing model, this study used release 4 of the ABCD data, from baseline to the 3rd annual follow-up wave. For details on data processing, such as the procedure for dealing with missing values, see SI Appendix S1 and Table S1. Inheriting fundamental concepts from the literature on “brain age” [26], “puberty age” was computed using supervised machine learning. The model aimed to learn the relationship between physical and hormonal measurements of puberty (specifically, each PDS item, DHEA, and TST levels) and chronological age, separately in males and females. To remove the potential impact of familial relations and repeat assessments of each individual, the sample was first stratified to randomly keep only a single observation for each family (i.e., across waves and siblings). Additionally, to ensure the model was trained on a typically developing sample; using the CBCL DSM-oriented scales (consistent with DSM diagnostic categories), we excluded individuals above the threshold (symptom’s score >60) for affective, anxiety, somatic, oppositional defiant and conduct problems, as well as ADHD. The remaining participants who had symptom scores <60 were included in the assessment of the performance of the puberty age model (typically developing (TD) sample *N* = 4949 (2439 females)). The model used a train, validation, and test split in which a subset of the TD sample (90% of TD) was used for training and validation (hyperparameter tuning) of the model. Thereafter, the optimal model was used to predict chronological age from pubertal measurements in an independent test sample (10% of TD). This procedure was repeated over 10 folds to provide out-of-sample age predictions for the complete TD sample. In addition, the trained model on the complete TD sample was used for age prediction in the non-TD sample. The effect of age was subsequently regressed from all predictions to adjust the bias created by regression toward the mean (RTM) [38]. For more details, see SI, Appendix S3, Figs. S2 and  S3. The bias-adjusted prediction of an individual’s chronological age from pubertal measurements is termed “puberty age”. Further, the residuals of the prediction model (after subtracting chronological age from puberty age) are referred to as the “puberty age gap”, which we use as a dimensional measure indicative of relative pubertal timing. A positive puberty age gap is interpreted as a sign of earlier pubertal development compared to an age and sex-matched group, while a negative gap reflects relatively delayed pubertal development (see, Appendix 1, Fig. S1).

We implemented a generalized additive model (GAM) for the prediction of age (response variable) from multivariate pubertal measurements (hormones [TST, DHEA] and/or each of the PDS items). As GAM fits smooth nonlinear curves in the form of spline functions, it is expected to outperform commonly utilized linear methods of pubertal timing measurement, given the nonlinear relations previously reported [30]. Inner loop validation and hyperparameter tuning were performed by a grid search for optimal regularization penalty on each term (i.e., using GAM) to minimize the estimated prediction error in the training sample (generalized cross-validation (GCV) score). The whole available sample was used for testing (*N* = 9919 (4725 females)). First, outer loop cross-validation with 10 folds was used to test the model for the TD sample. In other words, we divided the TD sample into 10 folds where each fold contains 10% of the TD sample. After this, the best parameters from the grid search for GAM were used to train a model on 9 folds and test in one remaining fold. This process was repeated 10 times and the accuracy of the model was calculated as the average for these 10 times. Additionally, a model was trained on the complete TD sample and tested on the non-TD sample to provide puberty age estimates for the complete (TD and non-TD) sample. The complete set of calculated puberty age measures were adjusted for the RTM age bias prior to subsequent analyses.

To understand the contribution of different indices to the measurement of pubertal timing, we implemented three alternative models to estimate “puberty age”. The first approach only used hormones (DHEA and TST) as features to predict chronological age, the second only used PDS items, and the third combined hormone and PDS items. The predictions of age were named *hormonal* puberty age, *physical* puberty age, and *combined* puberty age, respectively. In all three models, the residuals of age prediction indexed the “puberty age gap”, which reflects pubertal timing. We also used the partial dependence function in GAM models that can reflect the importance of each feature in the combined puberty age model (for more detail, see SI, Appendix S4 and Fig. S5).

#### Comparison of “puberty age” models

We compared the performance of the three alternate puberty age models using Pearson’s correlation between predicted and chronological age and mean absolute error (MAE) averaged over the complete sample (TD and non-TD). Additionally, we used a non-parametric paired *t*-test (Wilcoxon signed-rank test) on the absolute errors of model predictions to statistically compare the performance of different models.

#### “Physical puberty age” compared to a linear pubertal timing model

The current study also compared the out-of-sample performance and accuracy of the physical puberty age model to the commonly employed method of linearly regressing age from the average PDS score [19, 22]. In order to draw a comparison between the new model and the traditional linear approach, the performance of the physical puberty age model was contrasted with the traditional approach. The physical model was selected (rather than the hormonal or combined model) to ensure a fair comparison of two approaches that measure pubertal timing from the same input features (i.e., PDS). In order to conduct this comparison, a linear regression model was used to regress chronological age from the total PDS score (train) and used the fitted model coefficients in unseen data to measure timing (test). Similar to the puberty age model design, a 10-fold cross-validation design was used to measure traditional pubertal\ timing for the whole sample. This linear model provided an implementation of the traditional model in an out-of-sample prediction paradigm. Model performance was assessed based on the out-of-sample prediction accuracies (quantified by the absolute error of predictions). Similarly, we used the “Wilcoxon signed-rank test” to investigate the statistical differences in model performance.

#### “Puberty age gap” associations with mental health problems

We used linear mixed-effect models (LMM) to investigate associations between each alternate “puberty age gap” measure and different dimensions of mental health problems, in males and females separately. The following formula was tested for each syndrome dimension (i.e., Anxious/Depressed, Withdrawn/Depressed, Somatic Complaints, Social Problems, Thought Problems, Attention Problems, Aggressive Behavior, and Rule-breaking Behavior), as well as three broad scales including total problems, externalizing, and internalizing problems. Sample size for this analysis was *N* = 9919 (4725 (females)).

#### Mental health problems—puberty age gap + age + (1|site)

Age was included as a confound (fixed effect) and the data collection site was modeled as a random effect. We corrected for multiple tests controlling for False Discovery Rate (FDR) at 5% and reported the FDR corrected *p*-values in the results. The different “puberty age gap” LMMs were compared based on the Akaike Information Criterion (AIC) and a cut-off of 2 was used to indicate evidence for a better model, i.e., the model with an AIC that is at least 2 units smaller is considered a comparatively better model. Furthermore, to investigate whether associations between pubertal timing and mental health problems differed by age, we investigated the interaction effect of age and puberty age gap in predicting mental health problems using the following LMM:

#### Mental health problems—puberty age gap + age + puberty age gap: age + (1| site)

Finally, given the known association of pubertal timing with BMI, SES, and race/ethnicity [21], supplementary analyses repeated primary models while accounting for these variables as covariates (see SI, Appendix S5 and Table S1, for details of these analyses). This approach avoided potential complexity in our primary analyses due to the collinearity of confounding variables with our main variables of interest.

## Results

### Accuracy of puberty age models

**Fig. 1:**
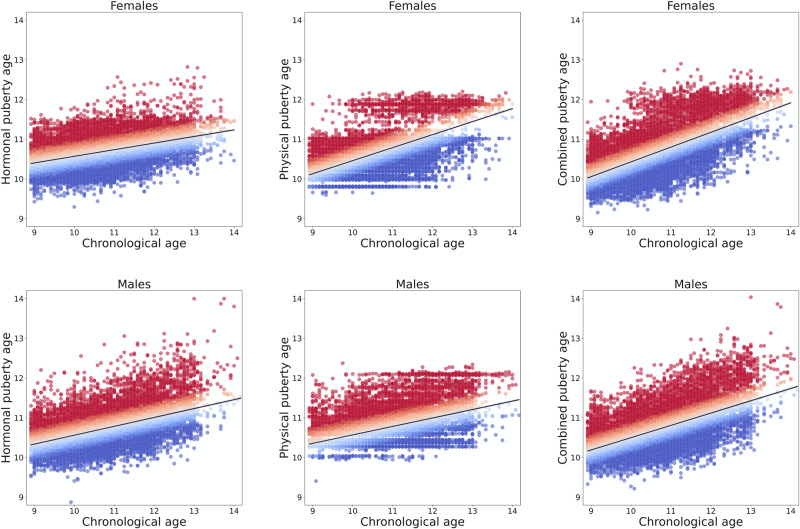
Performance of puberty age gap models. For all three models (combined, physical development and hormonal), the prediction of the age of each individual is plotted against chronological age (in years). Red dots indicate individuals with a positive puberty age gap (early timing) and blue dots indicate individuals with a negative puberty age gap (late timing).

All three models were able to provide significant and accurate out-of-sample predictions of age (Table 1, Fig. 1). We assessed the difference between the absolute errors of model predictions to statistically compare the performance of different models by a non-parametric paired *t*-test (Wilcoxon signed-rank test). The physical model was found to be a better predictor of age than the hormonal model in females (*p* < 0.001), while there was no difference between the performance of the hormonal model compared to the physical model in males (*p* > 0.05). Across both sexes, the combined model explained the largest degree of variation in chronological age and was more accurate as compared to the hormone and physical models (*p* values < 0.001). Refer to SI for the correlation between these three models (Appendix S2 and Fig. S1).

**Table 1:** Comparison of the out-of-sample prediction performance of alternative puberty age gap models.

	Females		Males	
	*r*	MAE	*r*	MAE
Combined puberty age	0.58	0.65	0.54	0.67
Physical puberty age	0.37	0.76	0.45	0.72
Hormonal puberty age	0.55	0.67	0.43	0.72

### “Puberty age” vs. linear model

Comparing the out-of-sample prediction performance of the physical puberty age with the (commonly used) linear model of pubertal timing (based on Pearson’s correlation and MAE) showed that there was a difference between these models in predicting age in previously unseen data (see Table 2). Further, non-parametric statistical comparisons showed that the absolute errors between models were significantly different (*p* < 0.001) with the physical puberty age model having a significantly lower prediction error in both males and females.

**Table 2:** Comparison of the out-of-sample prediction performance of the linear pubertal timing model and the physical development puberty age model.

	Females		Males	
	*r*	MAE	*r*	MAE
Linear pubertal timing	0.49	0.72	0.32	0.78
Physical puberty age	0.55	0.68	0.43	0.72

### Pubertal timing measures predicting mental health problems

**Fig. 2:**
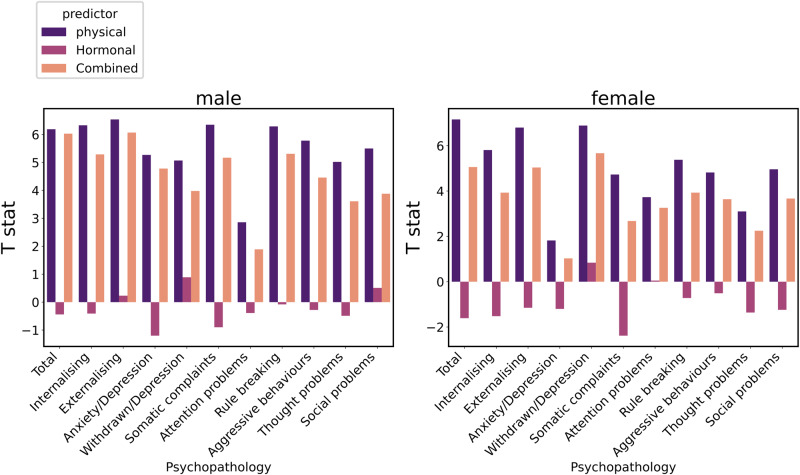
Bar plot showing the *T*-statistic from the prediction of each dimension of mental health problems from different puberty age models. Hormonal puberty age gap is not significantly associated with any dimension of mental health problems.

We investigated the association between each puberty age gap measure (from hormonal, physical, and combined models) and different dimensions of mental health problems (see Table 3, Fig. 2). Hormonal puberty age gap was not significantly associated with any mental health problems in either males or females. Physical puberty age gap, however, was significantly positively associated with all dimensions of mental health problems in males, and all dimensions except Anxiety-Depression in females. While similar associations were present for the combined puberty age gap, AIC differences indicated a better model fit for the physical puberty age gap in all instances. Finally, there were no significant interactions between age and any puberty age gap measure in predicting mental health problems in either females or males, suggesting that associations were stable across the examined age range.

**Table 3:** Associations between puberty age gap and mental health problems.

	Female						Male					
	Physical		Combined		Hormonal		Physical		Combined		Hormonal	
	*t*	AIC	*t*	AIC	*t*	AIC	*t*	AIC	*t*	AIC	*t*	AIC
Total	7.16***	**31,382.19**	5.06***	31,407.62	−1.61	31,430.59	6.19***	**34,308.27**	6.03***	34,330.51	−0.44	34,346.23
Internalising	5.81***	**31,145.29**	3.93**	31,163.47	−1.52	31,176.60	6.33***	**33,654.96**	5.29***	33,677.32	−0.41	33,694.64
Externalizing	6.80***	**30,528.71**	5.04***	30,549.40	−1.150	30,573.33	6.54***	**33,492.17**	6.07***	33,515.11	0.23	33,534.59
Anxiety_Depression	**1.82**	**26,535.61**	1.03	26,537.87	−1.20	26,537.47	5.27***	**29,000.46**	4.78**	29,019.26	−1.20	29,026.61
Withdrawn_depression	6.89***	**25,451.83**	5.67***	25,467.03	0.84	25,498.29	5.07***	**29,052.81**	3.98**	29,063.78	0.89	29,077.61
Somatic_Complaints	4.73**	**26,644.49**	2.68*	26,659.56	−2.38	26,661.07	6.35***	**28,592.79**	5.17**	28,619.53	−0.90	28,632.09
Attention_problems	3.73**	**26,187.00**	3.26*	26,190.28	0.05	26,200.82	2.86*	**29,291.46**	1.89*	29,296.84	−0.39	29,299.45
Rule_breaking	5.38**	**24,226.11**	3.93*	24,239.47	−0.72	24,254.27	6.29***	**26,982.27**	5.31**	27,005.78	−0.08	27,021.57
Aggressive_Behaviour	4.82**	**24,915.41**	3.64*	24,925.27	−0.51	24,938.20	5.78***	**28,625.15**	4.46**	28,646.70	−0.28	28,658.31
Thought_Problems	3.10*	**25,920.98**	2.25*	25,925.51	−1.36	25,928.69	5.02***	**29,131.42**	3.61**	29,147.13	−0.49	29,156.23
Social_Problems	4.96**	**23,988.88**	3.67*	24000.00	-1.24	24,011.85	5.50***	**26908.05**	3.88**	26923.08	0.51	26,937.86

Statistical significance is indicated by asterisks (*p < 0.05; **p < 0.01; ***p < 0.001; FDR corrected). The bold numbers reflect better model performance.

## Discussion

In this study, we utilized biological and physical pubertal features and used supervised machine learning to model pubertal timing and its association with mental health problems. Overall, the combined puberty age model predicted age more accurately than the two unimodal models (i.e., physical or hormonal models). We showed that our novel pubertal timing model (based on physical pubertal features) had better out-of-sample age prediction than a more commonly used linear regression-based measurement in both sexes. Findings also highlight that our new method of assessing pubertal timing was generally associated with mental health problems in males and females. We found that early pubertal timing (based on the physical and combined models) was consistently associated with various dimensions of mental health problems and could hence be a potential risk factor for general mental health problems.

Findings indicated that our novel normative puberty age models were able to provide out-of-sample predictions of age. This nonlinear modeling approach was found to be more accurate at predicting age than a linear model of pubertal timing that relies on linear modeling of the relationship between (average) pubertal stage and age. These results highlight the presence of nonlinear relationships between age and pubertal development, consistent with previous literature reporting the benefits of other nonlinear methods for calculating pubertal timing [21, 31]. Additionally, our examination of the collinearity between each of the PDS items showed that, although the items are significantly correlated, the correlations between them are <0.35 (for more details, see supplementary section 2). This suggests that each PDS item carries a significant amount of independent information, which our method is able to better capture through a multivariate design in comparison to mean PDS scores. Furthermore, our out-of-sample prediction provides an evaluation of the generalizability and replicability of study findings [39]. We presented how a model that was trained on 90% of the sample could provide accurate out-of-sample predictions in the remaining 10% of the sample. This provides the opportunity for future research to measure pubertal timing in studies with smaller sample sizes, by taking advantage of this pre-trained normative model. This could potentially alleviate some study biases inherent to small sample sizes.

Our novel method of assessing pubertal timing was also significantly related to mental health problems in adolescents. Across combined and physical puberty age models, we found that relatively early pubertal timing (i.e., positive puberty age gap) was associated with an increase in most dimensions of mental health problems. This is consistent with the maturation disparity hypothesis, whereby a mismatch between physical development and progression of emotional and cognitive development is purported to increase in those with early pubertal timing, which may account for difficulties navigating the complexities and challenges of this period and may thus result in greater risk for mental health problems [7, 4]. Although the prediction accuracy of the combined puberty age model was significantly better than physical puberty age, it had relatively weaker associations with mental health problems. Moreover, we did not find any significant associations between the hormonal puberty age gap and mental health problems in males or females. These findings are partially consistent with Barendse et al. [27, 40], who reported that pubertal timing measured from hormonal information (testosterone and DHEA) did not predict internalizing symptoms. Consistent with prior studies, these findings may suggest that psychosocial mechanisms have a larger role (in contrast to biological mechanisms indexed by hormones) in predicting mental health problems in those with early puberty [14]. Likewise, prior work has suggested that earlier physical development can impact social functions such as difficulty maintaining friendships with peers who mature later, and a tendency to associate with older adolescents who engage in more externalizing behaviors [12]. Of note, the associations between pubertal timing and mental health issues were of small effect sizes, and further research is needed to determine the clinical relevance of these findings.

While this study has strengths in its large sample size, use of hormonal assays, and physical measurement of puberty, as well as the implementation of a novel, generalizable method for assessing pubertal timing, there are limitations that should be addressed in future work. First, the age range of the sample used was restricted. While the correlations between some measures of puberty age and chronological age were modest, this is likely due to the limited age range within the dataset. Previous studies have shown how this constraint can affect correlation values in machine learning algorithms [41]. Further, a wider age range could have captured more variance in pubertal maturation (including the nonlinear relationship between age and pubertal development) and improved the out-of-sample model prediction of age. Relatedly, while we did not observe any interactive effects of age with pubertal timing in association with mental health problems, it is possible that such effects may be detectable across a larger age range. Further, different measures of the puberty age gap may also be differentially associated with mental health problems in earlier versus later adolescence. Second, this study focused on a limited number of pubertal hormones, and the omission of other hormonal features may have impacted the ability of the hormonal puberty age gap to predict age—particularly in females. Additionally, variations in the time for collecting hormones across the day could have impacted the performance of the hormonal age gap (although these were statistically accounted for). Thus, future studies could incorporate more detailed assessments of hormones to improve model prediction. While the current study opted for the parent-reported version of the Pubertal Development Scale (PDS) due to its reportedly higher reliability (as compared to self-report) within this specific age range, it is crucial to acknowledge that parent-reported measures can introduce inherent biases. These measures rely on subjective perceptions and parents are not always aware of their child’s specific stage of physical development. Consequently, it would be valuable for future research to replicate this analysis using clinician-rated pubertal development measures (which are suggested to be gold-standard [42]. Additionally, future work could look into the differences between self, parent, and clinician-rated physical measures of puberty and the sensitivity of pubertal timing (puberty age gap) evaluations to these alternative measures. Furthermore, we used the individual items of the PDS in our machine learning models rather than using summary measures (e.g. total score, total HPA and HPG scores, converted tanner stage). By considering each item separately, we aimed to uncover potential associations that might be masked by summary measures. Alternate approaches, such as creating separate models with items that map to HPA vs. HPG axes, may be important future endeavors.

In conclusion, the current study proposes a nonlinear puberty age model that facilitates generalizable investigations of pubertal timing in future studies. Our findings also highlight the importance of physical pubertal maturation, relative to hormonal changes, for mental health problems during early adolescence. This suggests that psychosocial mechanisms may play an important role in the relationship between early pubertal timing and mental health problems, which has implications for interventions aimed at reducing the risk of the emergence of mental health problems in adolescence.

### Supplementary information

Supplemental material
