# Supplementary material for: “Puberty age gap”: new method of assessing pubertal timing and its association with mental health problems
Source: Mol Psychiatry. 2023 Dec 5;29(2):221–8. doi: 10.1038/s41380-023-02316-4 (PMC11116096; doi:10.1038/s41380-023-02316-4)
Supplement: Supplementary file 1 — Supplemental material [file 41380_2023_2316_MOESM1_ESM.docx]

**Appendix S1**

# **Data cleaning and preparation**

The data for the ABCD study was collected and released across 4 longitudinal waves: baseline (baseline_year_1_arm_1, n = 11875), 1 year after baseline (1_year_follow_up_y_arm_1, n = 11225 ), 2 years after baseline (2_year_follow_up_y_arm_1, n = 10414), and for some participants ABCD also had information collected three years after baseline (3_year_follow_up_y_arm_1, n = 6251). We used the latest release of the ABCD data collection (release 4). To implement our normative models for pubertal timing, we first extracted both

hormonal and physical pubertal information from ABCD data sources. We used both collections of puberty hormones at each wave (Labels for DHEA: HORMONE_SCR_DHEA_REP1” and “HORMONE_SCR_DHEA_REP2”, and Testosterone: “HORMONE_SCR_ERT_REP1” and “HORMONE_SCR_ERT_REP2”). We processed the hormonal data for n= 38434 participants according to the procedure and guideline described in (Herting et al., 2021). Hence, the mean of repetitions 1 and 2 was computed. In the data cleaning process, there were n = 1331 participants who did not have information on caffeine intake or activity before collecting saliva samples. Another n =12098 did not have hormonal information of either DHEA and/or Testosterone. Finally, n = 25005 (n = 11034 females) participants remained for whom both DHEA and Testosterone were collected. Secondly, this study used sub-items of the Pubertal Development Scale (PDS) which contained growth spurt (PDS_1_P), body hair (PDS_2_P), skin change: (PDS_3_P), voice deep: (PDS_M4_P), face hair: (PDS_M5_P), breast development: (PDS_F4_P), and menarche: (PDS_F4B_P). Out of the remaining n=25005 with hormonal and physical pubertal data, another n =1888 were missing family ID information needed to familial relationships and n = 23117 (n = 11034 females) participants remained. For the remaining sample, hormonal outliers were replaced with a cap of 3 standard deviations from the mean. And finally, the hormonal and PDS data were quantile normalized to a Gaussian distribution. Finally, we only used this subset of the ABCD sample in subsequent analyses, e.g. model fitting and mental health assessments (Figure S1). To implement the puberty age model, we further filtered the sample to train the model. Specifically, first, a single time point of a single individual from each family was selected to remove the potential model bias to family relationships and repeated longitudinal measures (N = 9919 (4725 females)). Next, we used CBCL DSM oriented items (CBCL_SCR_SYN_INTERNAL_T, CBCL_SCR_SYN_EXTERNAL_T, CBCL_SCR_SYN_TOTPROB_T, CBCL_SCR_DSM5_DEPRESS_T, CBCL_SCR_DSM5_ANXDISORD_T, CBCL_SCR_DSM5_SOMATICPR_T, CBCL_SCR_DSM5_ADHD_T, CBCL_SCR_DSM5_OPPOSIT_T, CBCL_SCR_DSM5_CONDUCT_T, CBCL_SCR_07_SCT_T, CBCL_SCR_07_OCD_T, CBCL_SCR_07_STRESS_T, parent-report version) to extract the typical developing sample. This typical developing sample only included participants with all CBCL items scored below 60 (N = 4949 (2439 females)). The puberty age models were trained on this typical developing filtered sample to capture typical, healthy pubertal trajectories during development. This fitted model was then utilized to measure puberty age in the whole sample 9,919 (4,725 (females)). These values were combined with the cross-validated out of sample estimations of puberty age and were used for assessments of association with mental health. Additionally, to investigate the mental health problems from pubertal timing measures we used syndrome score from CBCL items including: cbcl_scr_syn_anxdep_t, cbcl_scr_syn_withdep_t, cbcl_scr_syn_somatic_t, cbcl_scr_syn_social_t, cbcl_scr_syn_thought_t, cbcl_scr_syn_attention_t, cbcl_scr_syn_rulebreak_t, cbcl_scr_syn_aggressive_t, cbcl_scr_syn_internal_t, cbcl_scr_syn_external_t, cbcl_scr_syn_totprob_t, while considering site as a random effect and age as a fixed effect. N= 9919 (4725 females)) remained for this association analysis.

Figure S1. Description of different puberty age models that use different information of puberty including hormones and physical measurement (PDS).


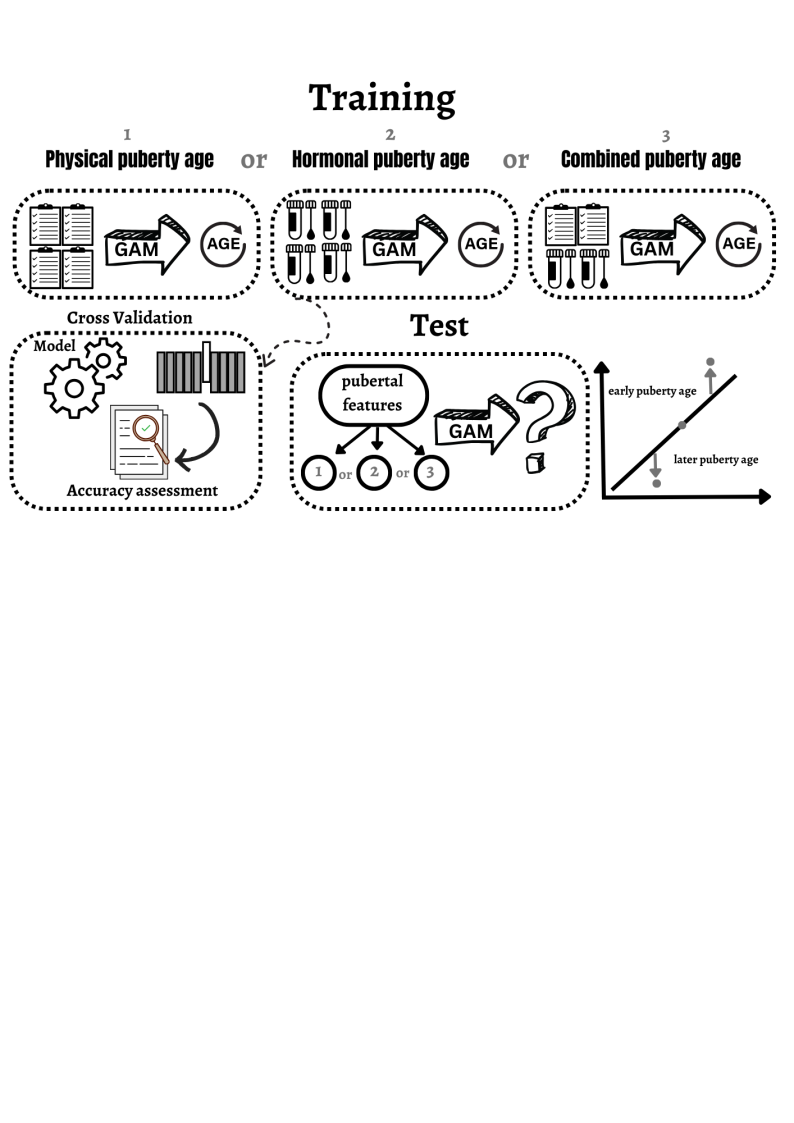


**Appendix S2**

# **Correlation of pubertal measures.** In this section, we have provided further information about the observed correlations between the three proposed models of puberty age gap (combined, physical, and hormonal), hormonal assays (DHEA and Testosterone), and PDS sub-items (skin change, growth spurt, body hair, menarche, breast development, voice deep, and facial hair) (see Figure S1). Results indicated that in males, the correlation between the combined age gap and the hormonal age gap was higher than the correlation between the combined puberty age gap and physical puberty age gap. In this sample, males are on average at an early stage of puberty where there is little variation in physical signs of puberty. In females, however, the correlation between the combined puberty age gap and the physical puberty age gap was higher than the correlation between the combined puberty age gap and the hormonal puberty age gap. This might reflect the wider variation in the physical signs of puberty in females at this age range. It could also reflect the fact that important female sex hormones were not investigated.

Figure S2. Observed Pearson’s correlations between measures of pubertal timing in males and

females.


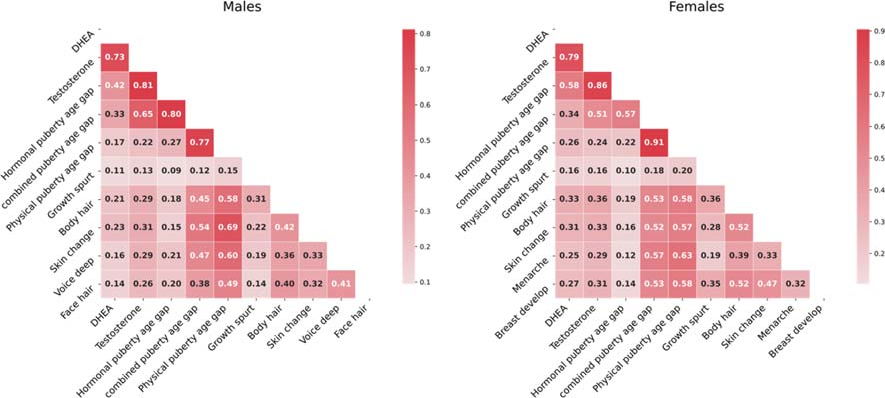


**Appendix S3**

**Correcting for regression to the mean.** This study removed the regression to the mean effect as a general model confound known to exist in regression problems dealing with some degree of uncertainty/randomness. Simply, when modelling a predictive relationship with a degree of certainty, a regression model will try to minimize the fit penalty by predicting closer to the population mean. In the context of our normative model, this shows up as an age bias in which older individuals tend to have a lower age prediction (relative to their true age) compared to younger individuals. This age bias can be explained by “regression towards [or to] the mean (RTM),” an elementary concept that has long been known in statistics (Davis, 1976; Gardner & Heady, 1973; James, 1973). In our method, RTM is expressed such that the younger children's age is typically overestimated, while older children's age is typically underestimated in all predictions. This bias can hence be corrected by a simple regression in which the age bias is removed from predictions. We investigated the correlation of the residual of the model (puberty age gap) with age before and after removing the regression to the mean. Figure 2 shows that the measures of a puberty age gap are initially biased and indicate accelerated timing in younger samples and delayed timing in older individuals, whereas this effect can be corrected by removing the RTM effect See Figure S3)

Figure S3. Performance of alternative puberty age gap models before removing regression to the mean correction


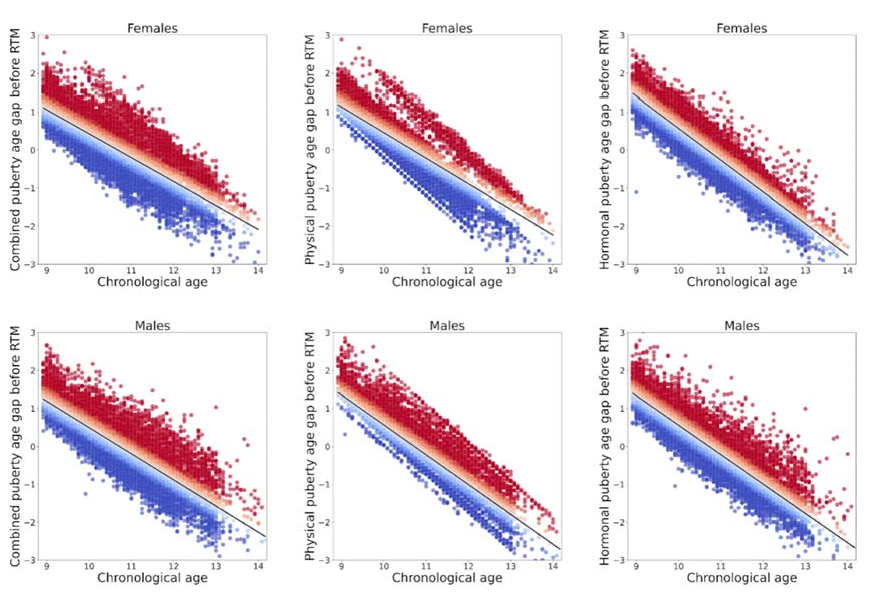


Figure S4. Performance of alternative puberty age gap models after removing regression to the mean correction


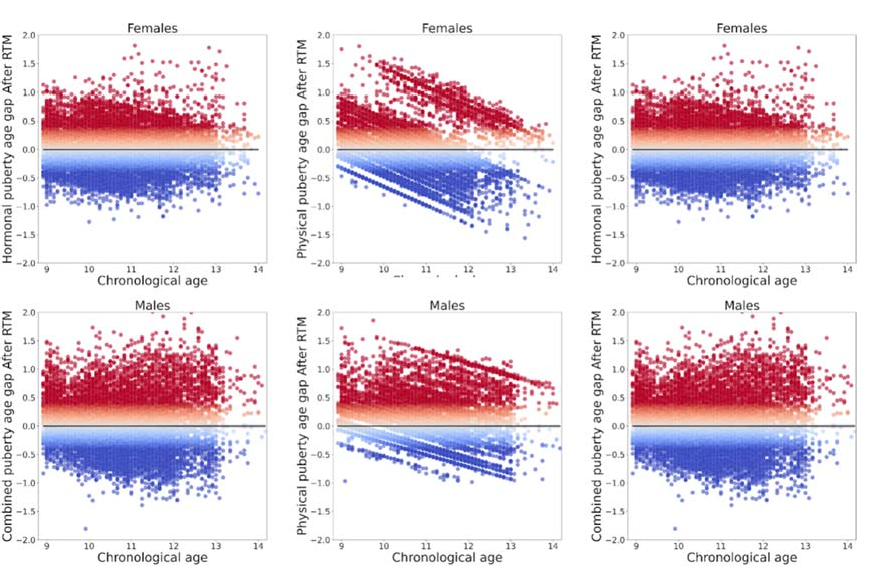


**Appendix S4**

# **Partial dependence functions.** In order to explore how different features of puberty contribute to the combined puberty age gap model, we used partial dependence (PD) functions in the Generalised Additive Model (GAM). A PD plot demonstrates the relationship between a single input feature and model predictions. They show how the predictions partially depend on the values of every input feature (See Figure S4). The results showed that Testosterone and DHEA (in both sexes) generally have a larger contribution to the combined puberty age gap compared to the physical measurements. Furthermore, these dependence functions indicate how many pubertal features express a nonlinear dependence with age. This verifies the assumptions of existing nonlinearities relating pubertal changes to chronological age. However, these partial dependence functions should be interpreted with caution as potential collinearities between the measures can confound the partial dependence functions to some extent.

Figure S5. Partial dependence functions describe the impact of every individual pubertal feature on the fitted GAM model.


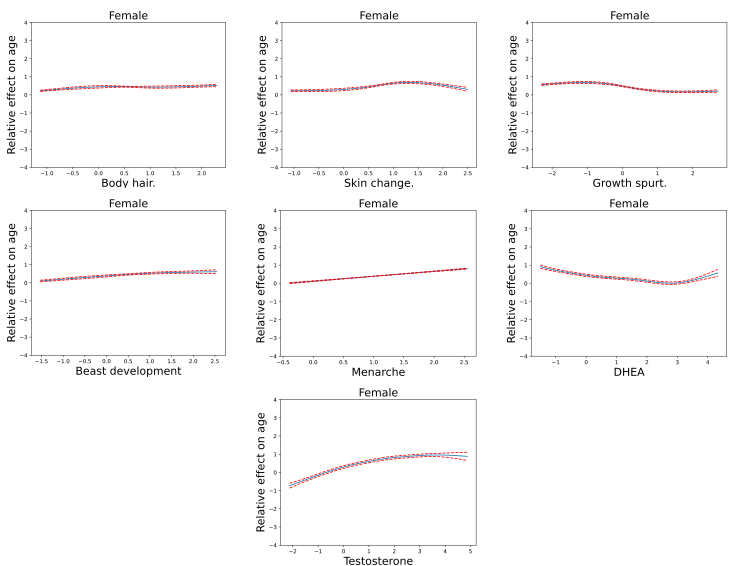


#

# **
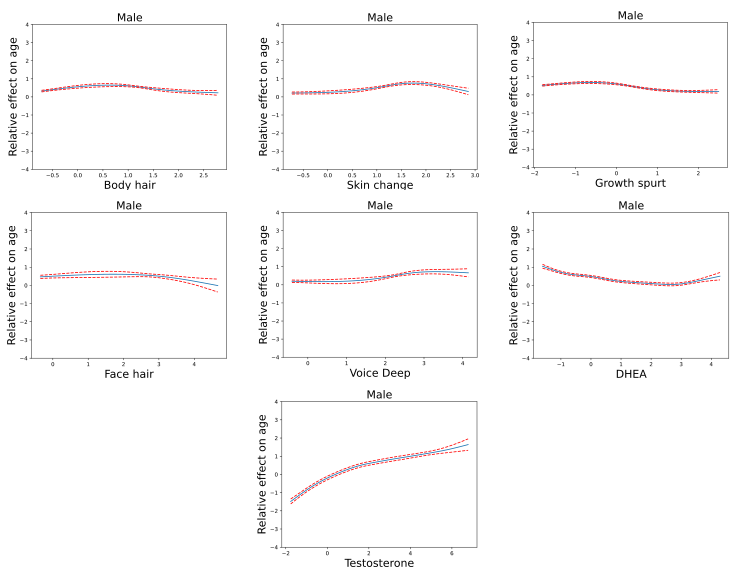
**

#

#

# **Appendix S5**

**Replication with a more detailed linear mixed model**

Known relationships of race, socioeconomic status, and BMI with both pubertal development

as well as psychopathology, and were therefore not included as covariates in the primary

model. For robustness testing and to make sure the result did not change in the presence of these covariates, the analysis was repeated with a more detailed model. We implemented a mixed effect model to predict psychopathology from pubertal timing measures (combined,

physical, and the hormonal puberty age gap) while considering the confounding effects of

race, socioeconomic status, and BMI. This more detailed model resulted in similar findings to

the simpler model presented in the manuscript. Hence, we have verified that while race, socioeconomic status, and BMI have had significant relationships with many psychopathologies, removing their effects as a confound did not change our study’s findings.

Table S1. Associations between puberty age gap models and psychopathology measured by linear mixed effect regression with considering SES, BMI and race as the fixed effects.

|  | Females | | | Males | | |
| --- | --- | --- | --- | --- | --- | --- |
|  | Physical | Combined | Hormonal | Physical | Combined | Hormonal |
|  | T-stat | T-stat | T-stat | T-stat | T-stat | T-stat |
| Total | 6.3*** | 4.1** | -1.4 | 5.9*** | 5.4*** | -0.3 |
| Internalising | 5.3*** | 3.3** | -1.2 | 6.0*** | 4.8*** | -0.2 |
| Externalising | 6.1*** | 4.0*** | -0.8 | 6.2*** | 5.1*** | 0.1 |
| Anxiety/Depression | 1.0 | 0.3 | -1.0 | 4.8*** | 4.1*** | -0.9 |
| Withdrawn/ Depression | 6.0*** | 5.0*** | 0.6 | 4.6*** | 3.3** | 0.8 |
| Somatic Complaints | 4.1** | 2.0* | -1.9 | 6.1*** | 4.8*** | -0.7 |
| Attention problems | 3.3** | 2.3* | 0.04 | 2.6* | 1.1* | -0.4 |
| Rule breaking | 4.8** | 3.2** | -0.8 | 6.0*** | 4.6** | -0.06 |
| Aggressive behaviors | 4.0** | 3.0** | -0.3 | 5.7*** | 4.1*** | -0.1 |
| Thought problems | 2.3* | 1.8* | -1.1 | 4.6*** | 2.9* | -0.4 |
| Social problems | 4.1** | 2.8* | -1.5 | 4.7*** | 3.2** | 0.4 |

Note: Statistical significance is indicated by asterisks (p<0.05: *; p<0.01: **; p<0.001: ***;FDR corrected). The bold numbers reflect better model performance.

Reference

Herting, M. M., Uban, K. A., Gonzalez, M. R., Baker, F. C., Kan, E. C., Thompson, W. K.,Granger, D. A., Albaugh, M. D., Anokhin, A. P., Bagot, K. S., Banich, M. T., Barch, D.M., Baskin-Sommers, A., Breslin, F. J., Casey, B. J., Chaarani, B., Chang, L., Clark, D.B., Cloak, C. C., … Sowell, E. R. (2021). Correspondence Between Perceived Pubertal Development and Hormone Levels in 9-10 Year-Olds From the Adolescent Brain Cognitive Development Study. *Frontiers in Endocrinology*, *11*(April 2020), 1–22. https://doi.org/10.3389/fendo.2020.549928
